# Psychological Impact of Spine Sarcoma on Wellbeing

**DOI:** 10.1002/pon.70546

**Published:** 2026-07-16

**Authors:** V. Williamson, G. Mawhinney, D. Cimatu, T. Land, J. J. Reynolds

**Affiliations:** ^1^ Institute of Psychiatry, Psychology and Neuroscience King's College London London UK; ^2^ Department of Psychology University of Bath Bath UK; ^3^ Oxford University Hospitals NHS Foundation Trust Oxford UK; ^4^ Nuffield Department of Clinical Neurosciences University of Oxford John Radcliffe Hospital Oxford UK; ^5^ Department of Immunology and Inflammatory Diseases University of Oxford John Radcliffe Hospital Oxford UK

**Keywords:** cancer, mental health, sarcoma, spine, wellbeing

## Abstract

**Background:**

Spine sarcomas are rare but debilitating cancers requiring extensive treatment with high morbidity. While physical outcomes are well studied, the psychological impact and support needs of patients remain poorly understood. The aim of this study was to qualitatively explore patient experiences of spine sarcoma on wellbeing and their perceived support needs.

**Method:**

Sixteen adult patients diagnosed or treated for spinal sarcoma at Oxford University Hospitals completed semi‐structured qualitative interviews. Data were analysed using thematic analysis.

**Results:**

Patients frequently experienced significant delays to diagnosis, often enduring severe, persistent pain and having requests for further diagnostic tests declined by healthcare providers. After diagnosis, many underwent extensive and invasive treatment, with prolonged hospital stays and reliance on considerable caregiving support after discharge. Emotional challenges were common, including anger, anxiety and social withdrawal, often compounded by chronic pain and reduced mobility. Patients also described unmet rehabilitative care needs, including fragmented aftercare and limited psychological provision.

**Conclusions:**

Findings underscore the urgent need for earlier diagnosis, enhanced clinical training, integrated psychosocial support, and coordinated long‐term survivorship care in spinal sarcoma.

## 1 Introduction

Spinal sarcomas are often highly debilitating, involving multiple vertebrae, epidural space with spinal cord compression, adjacent solid organs and the paraspinal musculature [1]. Treatment is extremely taxing, frequently requiring a multimodal approach involving surgery as well as radiation and/or chemotherapy, and—despite this—there is a relatively high rate of disease recurrence [1, 2]. Surgical treatment of spine sarcoma usually requires a complete resection without physical breach of the tumour capsule to gain maximal local tumour control [3, 4]. To achieve this, the planned surgical morbidity can be very high, including functional nerve root ligation. Nonetheless, there is the possibility that the tumour may return locally or with metastasis. Given its rarity, delays in diagnosis of spinal sarcoma are common and can result in considerably poorer patient outcomes [5, 6].

A further layer of complexity is added when the psychological implications of spinal sarcoma are considered. When a traumatic experience, such as cancer, occurs, it is critical to take account of the social, emotional and contextual factors that may influence adaptation [1]. However, despite the debilitating effects of spine sarcoma, the experiences of and challenges faced by patients with spine sarcoma has received limited research attention to date. Healthcare systems are increasingly recognising the importance of supporting mental health as a priority in cancer research and treatment [7, 8]. This is underscored by research that has found cancer patients feel that mental health care should be a core component of their treatment programme [8, 9]. Studies have also found that a considerable proportion of patients with cancer meet criteria for likely mental health disorders, such as post‐traumatic stress disorder (PTSD), depression, and anxiety [10, 11, 12]. Such psychological problems are highly comorbid and are associated with poorer quality of life, maladaptive coping, and substantial distress [13]. Mental ill health may be especially prevalent in patients with spinal sarcoma, particularly as the required treatment can call for extended hospital stays and prolonged isolation from family/friends, cause significant changes to one's appearance or mobility, and—despite invasive and aggressive treatment—there is high ongoing uncertainty about disease relapse [14, 15, 16].

As medical treatment advances, more patients are likely to survive cancer diagnosis and treatment. It is imperative that appropriate and accessible psychological support is available to ensure that ‘survivorship’ includes a good quality of life. Nonetheless, the provision of care in the aftermath of spinal sarcoma diagnoses is focussed on physical versus psychological [14]. Providing tailored guidance and support may enhance the wellbeing and quality of life of patients diagnosed with spinal sarcoma. However, the specific support needs of this patient group remain poorly understood [14]. Given the nature and complexity of spine sarcoma diagnosis and treatment, gaining deeper insight into patients lived experiences may help shape clinical practice and ensure that appropriate support, guidance and care are available to patients in future. To address this gap, we conducted semi‐structured qualitative interviews with patients with diagnoses of spine sarcoma to explore their experiences, impact of spine sarcoma on wellbeing and perceived support needs.

## 2 Method

The study received ethical approval from University of Bath Committee (5498–9020) and approval as a service evaluation from Oxford University Hospitals (OUH) (9129). All participants gave written informed consent for participation prior to taking part in any study assessments.

## 3 Participants

Between September 2024 and July 2025, *n* = 16 patients were recruited to the study. Patients were eligible for inclusion if they had attended OUH NHS Trust for spine sarcoma care and were aged 18 years or more. The following exclusion criteria were applied: unable to speak English, speech or hearing difficulties that prevented participation in a study interview, unwilling to provide informed consent. Of the 21 participants who agreed to be contacted by research team to take part in the qualitative interview, 16 consented to participate. No participants were excluded from the study, rather it was not possible to make contact with the remaining five participants.

### 3.1 Procedure

Participants were initially identified by the OUH clinical care team using study eligibility criteria. OUH is one of four UK centres delivering specialist spinal sarcoma service. The clinical team sought patient permission for their contact details to be passed to the research team. Given this agreement, patients were contacted by the study researcher (VW) with additional study information. Following informed consent, participating patients first completed assessments of their psychological adjustment and then participated in the qualitative interviews via MS Teams or telephone.

### 3.2 Assessments

To determine probable mental health disorders, the four item PHQ‐4 [17] was used to assess the common mental disorders (CMD) of anxiety and depression (cut off score ≥ 6). The three item Alcohol Use Disorders Identification Test (AUDIT‐C [18]) was used to assess likely alcohol misuse (cut off score of ≥ 8 to indicate high levels of alcohol misuse). The 18‐item International Trauma Questionnaire (ITQ) was used to assess probable PTSD and complex PTSD (CPTSD). A likely PTSD diagnosis is considered present if at least one symptom from each cluster (e.g., re‐experiencing, sense of threat, avoidance) was endorsed with a score of ≥ 2. A CPTSD diagnosis is made if the PTSD criteria are met, as well as at least one symptom from each of the DSO clusters (e.g., negative self‐concept, affective dysregulation, disturbances in relationships) [19, 20]. Demographic information was collected from all patients. Psychological outcome measures were used to describe the sample.

#### 3.2.1 Qualitative Interview Schedule

Interviews were carried out by VW by telephone or MS Teams and lasted an average of 66 minutes (SD 22.0). Any potentially identifying participant information was removed from interview transcripts and participant contact details were destroyed by the study researcher following the interview. The interview schedule was developed based on the research questions and previous studies exploring the lived experience of cancer patients (Supporting Information S1). The interview focussed on patient experiences of spine sarcoma diagnosis and treatment and its impact on wellbeing and daily functioning. Interview questions were open‐ended to encourage participants to recount in detail their subjective experience [21, 22]. Interviews were audio‐recorded with consent and transcribed verbatim. Interview audio files were destroyed following transcription.

### 3.3 Analysis

The Nvivo 12 software was used to facilitate analysis. An inductive thematic analysis approach was used and followed the steps recommended by Braun & Clarke [21]: repeatedly reading the dataset, generating preliminary codes, searching for and developing initial themes, and reviewing and organising themes. Data collection and analysis took place simultaneously to allow emerging topics of interest to be investigated further in subsequent interviews. A reflexive journal was kept throughout data collection and analysis by the study researcher who has expertise in qualitative methods (VW) to recognise the influence of the researcher's prior experiences and limit premature or biased interpretations of the data [23]. Peer debriefing was conducted, and feedback regarding data interpretation and analysis was sought from co‐authors (GM, JJR) with expertise in spine care and/or patient mental health.

## 4 Results

Of the 16 participants, 13 participants were male, with a mean age of 57.3 (SD 15.4; range 33–83 years). Two participants had received a diagnosis of spine sarcoma but had not yet begun treatment, three were actively undergoing treatment for spine sarcoma at the time of data collection, and 11 were post‐treatment. All participants identified as White British, approximately 60% (*n* = 9) reported living with a partner. Participants primarily reported working part time or full time or not working (e.g., due to retirement).

A number of participants met criteria for probable mental health problems on the psychometric measures, with 43.6% (*n* = 7) meeting criteria for CMD, 62.5% (*n* = 10) meeting criteria for likely alcohol misuse, and 12.5% (*n* = 2) meeting criteria for likely CPTSD.

### 4.1 Qualitative Findings

Three overarching themes were developed reflecting patients (i) experiences and impact of spine sarcoma diagnosis and treatment, (ii) experiences of recovery and navigating the ‘new normal’, (iii) perceptions of need for support in cases of spine sarcoma. Anonymised excerpts are provided to illustrate findings and demographic information are presented in Table 1.

**TABLE 1:** Participant demographic information.

Participant ID	Age at diagnosis	Gender	Occupational status
PID7	51 yrs old	Female	Employed
PID14	38 yrs old	Male	Employed
PID2	72 yrs old	Male	Retired
PID16	70 yrs old	Male	Retired
PID9	19 yrs old	Female	Self‐employed
PID5	43 yrs old	Male	Employed
PID11	57 yrs old	Male	Self‐employed
PID3	51 yrs old	Female	Employed
PID15	63 yrs old	Male	Self‐employed
PID1	37 yrs old	Male	Employed
PID13	52 yrs old	Male	Employed
PID6	78 yrs old	Male	Retired
PID12	34 yrs old	Male	Employed
PID8	57 yrs old	Male	Retired
PID10	56 yrs old	Male	Employed
PID4	68 yrs old	Male	Retired

#### 4.1.1 The Psychological Impact of Diagnosis and Treatment

For the majority of patients, their experiences of spine sarcoma initially began with pain, often experienced in their neck, back, legs or arms. This pain grew as time wore on, worsening to unbearable pain for some. However, despite intense pain for months (if not years) and growing concerns, many patients described being denied diagnostic tests or imaging by primary care. Patients described being repeatedly told by clinical care teams that their pain was “just” due to factors including arthritis, ageing, poor posture, or work in physically demanding roles.

Took about 18 months to get to the point of finding out that I had the tumour. It's quite a long period of time where… I was in pain…and trying to cope…They said I had a bit of arthritis…I would say to the physio… it's not getting any better…I got forwarded on to [a] musculoskeletal person. He basically said I needed to improve my posture…I put my form that I'd like to have a scan, that wasn't taken up…The pain continued. I was always on the phone to the GP… [telling them] it's a really intense pain in the back of my neck and my head and I can't sleep … I went back to my GP in tears said, “I can't manage this anymore.” [The GP said] do you want to pay for your own scan? I said yes. I want to [have a] scan. So, he rang up the [private] MRI department. I went the very next day.

(PID3, female)

Notably, after repeated failure to access necessary care, several patients described having little choice but to pay for their own imaging scans privately to understand the cause of their debilitating pain. Others described being repeatedly turned away by primary care until they had additional symptoms, such as limb numbness or incontinence, before they were finally sent for investigative tests.

[I had] a few issues with my back…I've been to the doctors a few times with it… But then last year, it progressively it got worse…. I went to the doctor's and said, look, my back's playing up again, but I've got other symptoms like I was getting pins and needles in the top of my thighs…And luckily, [the doctor] said…‘because of those symptoms, we can get you in for an MRI.’ [The doctor] was like ‘they don't like doing MRI scans at the minute.

(PID12, male)

Patients who experienced extensive delays to diagnosis described feeling vindicated—that their pain was valid and had an organic cause. Patients also described intense feelings of disappointment, anger and loss of trust towards healthcare providers. Several patients reported that if they had received their diagnosis sooner, they may have had more treatment options available, or their prognosis would have been better. The delays to diagnosis and their concerns being dismissed by healthcare providers reportedly contributed towards patient fears that the sarcoma may reoccur in the future.

It makes me think that maybe… if I'd had a scan earlier, it might have been caught sooner and it might have had a different projection as to the way they dealt with it or could have dealt with it. I was angry…I said to my doctor…that I'm really angry about this. And I can't believe that all these people have told me that there's nothing wrong with me.

(PID3, female)

#### 4.1.2 Impact of Diagnosis on Loved Ones

Patients described that receiving their diagnosis of sarcoma came as a considerable shock. Sharing the diagnosis with their families was a key challenge shared by patients, particularly if they had young children. Many recounted that their spouses/partners and children were very distressed by the news, with substantial questions and concerns. Having the opportunity to bring loved ones to pre‐operative consultations was experienced as helpful, allowing questions/concerns to be addressed. Several patients reported being part of a research trial where their consultation with the treating surgeon was video recorded. Patients found the opportunity to re‐watch the footage about their sarcoma and planned treatment, as well as share the recording with loved ones, very beneficial as they often could not remember key details of the consultation and were able to comprehensively share information with others.

[In] the pre‐op when I had quite a long chat…which [the surgical team] videoed. And it was explained to me all about the chordoma…they said about it could have been sitting there for about eight years…So that that was a bit of a shock as well because you think, well [why] wasn't it picked up earlier? … [It was filmed] with my agreement, but it was great because you know when you're told something like that, you can't take it all in properly. So… we could watch it afterwards… We sent it to all [our] kids… So that was… easier than sort of telling them really [and] then having lots of questions from them.

(PID4, male)

#### 4.1.3 Impact of Treatment

Many patients reported receiving treatment which involved highly invasive surgery to remove the sarcoma, often resulting in vertebrae resection, and nerve sacrifice or loss of bladder/bowel function in some cases. Several patients had to receive further post‐operative treatment, including proton therapy, often overseas or at other non‐OUH sites. Others received medications to control their tumour growth that could cause harmful birth defects, and they were therefore unable to have further children.

Patients who underwent substantial surgeries often woke in intensive care units (ICU) in significant pain and, notably, several reported vivid hallucinations on awaking as a result of the anaesthesia. These hallucinations were often frightening, persecutory and caused substantial distress. Yet no patients expressed being forewarned by their clinical care teams about this possible side effect. Patients described having to spend weeks or months in hospital recovering after surgery, some needing to relearn how to walk, move their arms or swallow. Many found this time in hospital incredibly isolating, particularly if they received care during the social distancing restrictions of the CV‐19 Pandemic. Visits from family and friends were a key source of support during this time, although logistically this was challenging as many patients did not live locally, and the journey strained their social support networks.

I was in hospital a month, so that was that's quite a long time and it was quite an uncomfortable month… I couldn't swallow. I couldn't drink. I couldn't eat…It was horrible. That was painful… I had visits every day pretty much. That was important to me… Various members of my family visited. We had friends [locally] who [my family] could stay with and that was great. And my parents came, sisters came, and some friends came too…. Having a visit every day, it just breaks the day up.

(PID13, male)

#### 4.1.4 Experiences of Recovery and Navigating the ‘New Normal’

On discharge, many patients described a lengthy recovery process. Practical support from loved ones was instrumental as multiple patients struggled with post‐operative mobility and ongoing pain. Rehabilitative after care from healthcare providers was frequently poor, with patients experiencing challenges accessing mobility aids, lack of referrals to pain management clinics despite chronic pain, and limited access to appropriate follow‐on care as primary care physicians or physiotherapists had little or no knowledge of spine sarcoma or chordoma. After care was often fragmented, with some patients required to travel long distances to OUH for follow up scans and blood tests, even when local facilities were available. Patients reported having unanswered questions or concerns about their recovery and difficulty contacting their surgical team after discharge.

All my scans have to be done in Oxford… I've got to go to Oxford for all of them, but I'm five minutes away from [my local hospital] … I can get all the scans done there… It just seems mad in this day and age that those scans can't be done [locally]… The operation…that was all great, but the [after] care now it just seems to have just tailed off…I've got a call with [my oncologist]. And I've got another telephone call with [my surgeon] … And I suggested that the three of us get together on a call, but I haven't been able to put that together either. And I just think it's so easy to do with modern communications these days. I could sit here on my laptop, talk to both the [doctors] at the same time…But nothing seems to be joined up…but at the end of the day, what can I do?

(PID11, male)

Patients who required additional surgeries (e.g., if bone grafts were unsuccessful) or experienced a substantial loss of daily functioning reported substantial adjustment difficulties. High levels of persistent pain and reduced mobility often led to leaving employment, as commuting or meeting the physical demands of their roles became unmanageable—contributing to a loss of identity and social isolation. These limitations also deterred participation in previously enjoyed activities, due to concerns about exacerbating pain or practical barriers (e.g., availability of seating, travel feasibility). Patients experiencing incontinence described feelings of shame, further reinforcing social withdrawal. Overall, these individuals reported difficulties reconciling with what they perceived as a new, less able‐bodied version of themselves.

When I had to give up work… I got to a point where… I'm isolating myself away from [old colleagues] because it's making me sad that I can't be [working]… So, I've stopped…meeting with them… my social circle has really shrunk… When I say I haven't really accepted the new me. It's not just the new disabled me, it's also the new me that doesn't [work] anymore…I’ve recently started seeing a psychologist privately to try and get into a place where I can work out who I am again because I don’t really know who I am at the moment… Which I think hasn't helped with my pain management because I haven't been in a good place in my head.

(PID7, female)

Difficulties coping following spine sarcoma diagnosis and treatment were not experienced by all patients. While also experiencing mobility problems and ongoing pain, several patients described that they were coping well and reported using specific cognitive avoidance strategies throughout their sarcoma journey. Patients described actively not wanting to know about their tumour or planned treatment following diagnosis. They reported refusing to look up their condition online, avoiding looking at scans and avoiding speaking about it with loved ones working in the medical field who might be knowledgeable. Many of these patients described that they had accepted and come to terms with the fact that their life was different post‐diagnosis/treatment and reported refusing to think about what functioning they had lost, maintaining a positive focus instead on what functioning they retained. Setting a clear recovery goal was also experienced as helpful.

[The doctor] mentioned the type of tumour… And I never have Googled it …because why would I want to read all the hideous things that it says about it? … [The doctor] said you are technically… disabled now. I remember looking over thinking, I do not accept that I am disabled…. I have always been independent… I suppose [I had an] acceptance of…things are not going to be the same after this surgery as they were before… But equally, I did not want to accept that I was going to be disabled or that I could not do stuff that I did before, and that, for me, made me determined to recover and not accept that my life was particularly diminished by any of this.

(PID8, male)

Notably, four patients reported being UK Armed Forces (AF) veterans and described that their military training and experiences had helped them to cope well while under stress—which they drew upon during sarcoma treatment and recovery. Similar to military operations where implicit trust of fellow service personnel is central to operational success, these patients described placing similar trust in their surgical team—a factor they regarded as central to their positive post‐operative outcomes.

Say you are flying with a pilot, and you have to trust the pilot [that] he’s gonna do his job properly…I think that was transferred to me to [the surgeon] and thinking, okay, this [surgeon] is like my night flying pilot. I trust him to do what he is going to do.

(PID8, male)

Despite experiencing psychological and physical health difficulties, all patients described experiences of post‐traumatic growth. Post‐traumatic growth included increased appreciation for loved ones and perceived improvements in one's ability to empathize with others (particularly those with cancer). Having met others who were more severely affected by treatment or did not survive, many patients expressed feeling fortunate to have survived, often noting that others had faced worse outcomes.

I look back… I think I can't complain…. When I was [getting treatment]… a couple of parents come in…and the mother came back with the daughter and she'd had a brain tumour. And you look at this little girl and she was seven years old. She had no hair. She had a patch because she had to have an eye removed…And I look at that, and I just remember that. And I just think I felt I almost felt like a fraud being there…And you know, there's me and I'm just complaining because… I've [had surgery and] got problems going to the toilet. I think really?

(PID15, male)

### 4.2 Perceptions of Need for Support in Cases of Spine Sarcoma

A primary pre‐treatment concern expressed by patients related to expectations regarding physical function post‐treatment (e.g., running, lifting). In retrospect, patients valued this information being provided ahead of surgery in a straightforward manner by their clinical care team as it gave them time to come to terms with planned morbidity. Access to guidance about the financial support available through government grants or charitable organisations also helped to relieve significant stress. Workplaces that were supportive, offering light duties or extended sick leave, were valued. A number of patients described seeking formal psychological support—often privately due to long waiting times—to help them to process their sarcoma diagnosis, treatment and recovery, as well as to support their management of chronic pain, low mood and adjust to physical limitations.

We've got to go and sort out finances and stuff and…see what we can claim for…we've got to meet with a lady next week…to see…if there's anything we can claim… I just didn't want to…be stressing with not bringing home what I normally bring home….I didn't want my wife to have to deal with all of that…[I work for] an American company. So, they don't pay sick pay in America…. So unfortunately…I'm only getting like…15 days fully paid…it’s not long… I've got a family and…stuff needs to be in place.

(PID12, male)

As rehabilitative after care was often disjointed, inadequate or misinformed, patients reported a need for a comprehensive information ‘pack’ that could clearly brief their primary care providers about their spine sarcoma diagnosis and ongoing recovery needs and ensure necessary referrals (e.g., to pain management clinics, incontinence services) were made. Given the difficulty in accessing ongoing care, patients also highlighted the value of a vetted list of trusted and knowledgeable care providers (e.g., physiotherapists, psychologists) who could support their recovery as these professionals were reportedly hard to find. Furthermore, poor collaboration between departments involved in their care (e.g., primary care, oncology, orthopaedics) often resulted in conflicting advice from different specialities. Patients felt that a more coordinated, holistic approach—or the appointment of a dedicated care coordinator—would have simplified navigation of the complex NHS care pathways and improved both treatment and recovery.

My biggest disability is the pain that I've got… I haven't been to a pain clinic…Surgeons and oncologists and GPs all have got different views about what medication I could try….I haven't ever seen anyone to…help psychologically with my pain, which I think would be helpful.…I saw the incontinence service because of the no bowel and bladder sensation stuff… I just hope that people who are [less familiar with the NHS] would get pointed in their direction because…I went and found that [service] for myself… it's quite difficult I think because surgeons are surgeons and they know their surgery, and the oncologist wants to talk about your cancer and then you're…reliant on your GP. And…getting a hold of a GP now is almost impossible… Having some sort of care coordinator…that can … think about all the bits of you, not just the bit that's their speciality and think about how one thing effects something else. That that makes big difference.

(PID7, female)

## 5 Discussion

This study aimed to explore the experiences of patients with spine sarcoma, focussing on the impact of diagnosis and treatment on wellbeing and their perceived support needs. We identified three key themes: the lived experience of the diagnosis and often intensive treatment, coping with recovery and adapting to a ‘new normal,’ and perceptions of the support required for individuals with spine sarcoma.

### 5.1 Clinical Implications

Patients frequently experienced significant delays to diagnosis, often after enduring severe, persistent pain and having requests for further diagnostic tests declined by healthcare providers. Many patients sought help from multiple providers, including GPs, physiotherapists, chiropractors and Accident and Emergency (A&E) doctors. This finding is consistent with previous UK studies with primary bone cancer patients typically require eight visits to various healthcare providers before receiving an accurate diagnosis [24]. These findings highlight gaps in healthcare providers' training and knowledge about spine sarcoma, as well as opportunities for healthcare providers to intervene earlier and improve access to timely care and patient outcomes.

The qualitative approach used in this study allowed for an in‐depth examination of patients' emotional responses and appraisals following diagnosis and treatment. For many, the diagnosis was described as a profound shock, and their loved ones also reportedly experienced significant distress. Patients often underwent extensive and invasive treatment, with prolonged hospital stays for recovery, often requiring considerable caregiving support after discharge. Several patients described marked emotional challenges, including shock, anger, anxiety, shame and social withdrawal—compounded by chronic pain and reduced mobility. This is consistent with the broader psycho‐oncology literature [25, 26] and may highlight potential targets for future interventions in spine sarcoma care. As prior life experiences can influence coping responses [27, 28, 29, 30], these factors may warrant consideration during psychological assessment and support for sarcoma patients. Patients in this study who reportedly coped well with their new physical limitations tended to accept the changes and focussed on their retained functioning. This could potentially suggest that Acceptance and Commitment Therapy (ACT) may be particularly beneficial in this population. ACT has been shown to significantly improve acceptance, depression, anxiety and quality of life in cancer patients by supporting them to manage negative emotions while maintaining a goal directed positive outlook [31]. Notably, patients who had the opportunity to share a video recording of their pre‐operative consultation with their surgeon reported that this was invaluable in helping them communicate the diagnosis to their families and manage subsequent questions and concerns. Future research in rare and complex cancers should consider integrating technology‐based solutions to better support patients and families in understanding both diagnosis and treatment [32, 33].

In terms of post‐treatment adjustment, patients consistently reported difficulties accessing high quality aftercare. Many lived with chronic pain without referrals to relevant pain specialists and struggled to find clinicians familiar with spine sarcoma. There was reportedly a clear need for improved communication between clinicians across departments and hospitals to coordinate care and prevent conflicting advice, as well as for a vetted list of appropriate follow‐up support services (e.g., physiotherapists, psychologists) that patients could access if needed. The supra‐regionalisation of rare disease treatment concentrates medical and surgical expertise but dilutes local support options. In the UK, opportunities exist to develop a more structured hub model, which is currently delivered in an ad hoc manner at best, and in many areas is entirely absent. Cancer survivors who have access to long term follow up care by their oncology team or dedicated clinic have been found to have better long‐term physical and psychosocial health outcomes than those unable to access this care [34]. That patients in this study reported a need for improved post‐discharge care aligns with the NHS’ future goal to providing cancer patients with a clear, tailored package of support following primary treatment [35]. Targeted efforts are required to ensure spine sarcoma patients have access to essential services and resources to optimise their quality of survival.

### 5.2 Study Limitations

This study has several strengths and limitations. A key strength is the use of qualitative methods, which enabled an in‐depth exploration of patients' experiences within a modest sample size, allowing for detailed analysis of the data. Participation was carried out remotely (e.g., online or telephone) by a researcher (VW) independent of the OUH clinical care team. Participation was anonymous and confidential, which may have helped patients to share their lived experiences openly. Limitations include the use of opportunity sampling and the limited demographic diversity of the sample, with most participants being male and White British. However, these demographic characteristics are broadly consistent with other studies of spine sarcoma carried out in this geographical area and NHS Trust [36]. A future large‐scale international investigation of patient experiences of spine sarcoma care and its impact on wellbeing would be valuable to explore how these findings compare across healthcare systems and patient populations globally.

## 6 Conclusions

Despite these limitations, the present study makes several contributions to the literature. First, these findings extend our understanding of the lived experience of spine sarcoma by moving beyond the physical treatment to highlight the underexplored psychological dimensions. Second, the findings underscore the perceived impact of diagnosis and treatment on wellbeing, offering insights that may help organisations and clinical care teams deliver more targeted support. Finally, the study highlights significant gaps in aftercare, emphasising the need for improved long‐term pathways to enhance access to services and optimise quality of survivorship.

## Funding

This research was funded by the Bone Cancer Research Trust.

## Ethics Statement

This study was reviewed and approved by Oxford University Hospitals (Service Evaluation OUH 9129) and University of Bath (REC no 5498–9020).

## Conflicts of Interest

The authors declare no conflicts of interest.

## Supporting information


Supporting Information S1

## Data Availability

The data that support the findings of this study are available on request from the corresponding author. The data are not publicly available due to privacy or ethical restrictions.
