# Supplementary material for: Psychological Impact of Spine Sarcoma on Wellbeing
Source: Psychooncology. 2026 Jul 16;35(7):e70546. doi: 10.1002/pon.70546 (PMC13373986; doi:10.1002/pon.70546)
Supplement: Supplementary file 1 — Supporting Information S1 [file PON-35-e70546-s001.docx]

**Supplementary Material 1 - Indicative guide for in-depth interviews**

**Topics to explore in the interview**

- How would you describe your physical health over your lifetime?
  - Can you tell me about your physical health *before* your sarcoma diagnosis at OUH?
  - Can you describe any key turning points in your health?
- When did you receive your sarcoma diagnosis at OUH?
  - What was this experience of diagnosis like?
  - How was the condition and treatment first explained to you at OUH?
  - What were your reactions at the time?
  - Did you have any questions/concerns at this stage?
  - Was there anything you think could have been done at this stage to better support you at OUH?
- Can you tell me about receiving treatment for your sarcoma at OUH?
  - What treatment did you receive at OUH?
  - What was this treatment like?
  - How long did it take from your diagnosis to receive treatment at OUH?
  - Do you have any further treatment planned at OUH? If yes, can you describe this.
  - What impact has receiving this treatment from OUH had on you?
  - Did you have any concerns/questions while you were receiving treatment at OUH?
  - Was there any more information you would have liked to have had?
  - Could anything have been done differently here?
- Can you describe what your family/friends thought about your diagnosis/treatment at OUH?
- Has your diagnosis/treatment at OUH had any impact on your work?
- Have you spoken to any other people who have received a similar diagnosis/treatment at OUH?
- Is there anything about your health or experience at OUH that we’ve left out?
- What are your feelings about this interview and all that we have covered?
